# Perineal herniorrhaphy with pedunculated tunica vaginalis communis in dogs: Description of the technique and clinical case series

**DOI:** 10.3389/fvets.2022.931088

**Published:** 2022-08-04

**Authors:** Tatsuya Heishima, Kazushi Asano, Kumiko Ishigaki, Orie Yoshida, Naoki Sakurai, Kazuyuki Terai, Mamiko Seki, Kenji Teshima, Shigeo Tanaka

**Affiliations:** ^1^Laboratory of Veterinary Surgery, Department of Veterinary Medicine, College of Bioresource Sciences, Nihon University, Fujisawa, Japan; ^2^Laboratory of Veterinary Anesthesiology & Respiratory Research, Department of Veterinary Medicine, College of Bioresource Sciences, Nihon University, Fujisawa, Japan

**Keywords:** dogs, herniorrhaphy, perineal hernia, Trendelenburg position, tunica vaginalis communis

## Abstract

**Background:**

Perineal hernia (PH) in dogs is caused by the separation of the anal septal muscles and the displacement of pelvic/abdominal organs under the perineal skin. Reconstruction of the pelvic septum by surgical repositioning is the only curative treatment.

**Objectives:**

To describe the procedure and outcomes of surgical repair using the pedunculated tunica vaginalis communis (TVC) for PH in dogs.

**Methods:**

Intact male dogs diagnosed with PH were included in this study. For surgery, each dog was positioned in the Trendelenburg position. Castration was performed with the open technique, followed by colopexy and cystopexy *via* laparotomy. The remaining bilateral TVCs transposed to the opening of PH were used for the perineal herniorrhaphy. Intraoperative findings, complications, and outcomes were evaluated and recorded.

**Results:**

Eight dogs [median age 10.5 years (range, 9–13 years); median body weight 4.9 kg (range, 1.6–12.3 kg)] were treated using the TVC surgical technique. Perineal herniorrhaphy with the TVC was feasible in all dogs. The median operation time was 105.5 min (range, 46–149 min) in unilateral PH, and 92 and 122 min in two dogs with bilateral PH. Short-term postoperative complications during hospitalization did not occur in six dogs, whereas the residual two dogs had a temporary local infection as a minor complication. Postoperative recurrence occurred in one dog (13%) on postoperative day 136.

**Conclusions:**

Our study suggests that the herniorrhaphy technique using the pedunculated TVC is an alternative option for the repair of PH in dogs.

## Introduction

In dogs, perineal hernia (PH) is caused by the separation of the anal septal muscles and the displacement of pelvic/abdominal organs under the skin of the perineum due to atrophy of the levator ani and coccygeus muscles; these muscle groups make up the pelvic diaphragm. PH most often occurs in middle-aged and elderly male dogs, of which most are intact males (1). Furthermore, as castration may prevent PH (2), muscle atrophy is thought to be caused by abnormal muscle metabolism and muscle fibrosis due to hormones produced from the testis (3) and the overproduction of relaxin associated with benign prostatic hyperplasia (4); however, a definitive pathogenesis of PH is still unclear.

Castration is recommended during perineal herniorrhaphy in intact male dogs, and reconstruction of the pelvic diaphragm components by surgical repositioning is the only curative treatment (2, 5, 6). In brief, anatomical herniorrhaphy is carried out to close the hernial foramen with the external anal sphincter muscle, levator ani muscle, coccygeus muscle, internal obturator muscle, and sacrotuberous ligament (1). However, this traditional technique is difficult and fails to close the hernial foramen when atrophy of the pelvic diaphragmatic muscles is severe. In addition to the traditional technique, various surgical procedures have been developed for reconstruction using autologous tissues, including the internal obturator muscle (7, 8), semitendinosus muscle (9), fascia lata (10), and sacrotuberous ligament (11). Internal obturator muscle transposition herniorrhaphy is the most common among them: however, previous studies have reported postoperative complications, including seroma, infection, tenesmus, dyschezia, fecal impaction, stranguria, and urinary incontinence, most of which resolved within a few days (7, 8). More than one year after the operation, 51.2%−93% of the dogs were free of complications (7, 8). The one-year post-surgical recurrence rate was 0–27.4% (7, 8).

In recent years, perineal herniorrhaphy using the extracellular matrix (ECM) has been performed, and the use of small intestinal submucosa (12–14) and tensor fascia lata (10) has also been reported. These materials are used in veterinary medicine not only for the treatment of PH but also for orthopedic diseases such as tendon rupture (15), deep periodontal bone loss (16), and corneal ulcer (17, 18). Previous studies have reported that the tunica vaginalis communis (TVC) graft could be implanted for perineal herniorrhaphy in dogs (19, 20). However, the strength of the free graft could reduce over time due to the absence of blood supply. We hypothesized that the pedunculated TVC graft is more effective for perineal herniorrhaphy than the free graft due to the preservation of blood supply. Therefore, this study aimed to describe the surgical procedure of herniorrhaphy with a pedunculated TVC graft after open castration for canine PH and evaluate the intraoperative and postoperative findings of canine patients treated using this procedure.

## Materials and methods

### Animals

Eight intact male dogs diagnosed with PH were referred to our animal hospital. In the first evaluation, the clinical symptoms of PH in all dogs were confirmed. In addition, a rectal digital examination, complete blood count (CBC), serum chemistry, and abdominal radiography were performed for the diagnosis of PH. All dogs underwent perineal herniorrhaphy with the pedunculated TVC graft. Prior to the first evaluation, informed consent was obtained from all owners for all procedures. In addition, all tests and procedures were approved by our institutional review board in compliance with the ARRIVE guidelines.

### General anesthesia and perioperative management

As premedication, 1.0 mg/kg of maropitant citrate hydrate (Cerenia^®^; Zoetis, Parsippany, NJ, USA) and 0.04 mg/kg of atropine sulfate (Mitsubishi Tanabe Pharma Co., Osaka, Japan) were administered subcutaneously. In addition, 0.1 mg/kg midazolam (Dormicum^®^; Astellas Pharma Inc., Tokyo, Japan) and 5.0 μg/kg fentanyl/0.25 mg/kg droperidol (Thalamonal^®^; Daiichi-Sankyo Propharma Co., Ltd., Tokyo, Japan) were administered intravenously (0.1 ml/kg). For the induction of general anesthesia, propofol (Mylan Seiyaku Ltd., Tokyo, Japan) was intravenously administered, and endotracheal intubation was performed. During the surgery, anesthesia was maintained with isoflurane (IsoFlo^®^; Zoetis), and artificial ventilation was performed with oxygen.

Continuous intravenous administration of 10–40 mg/kg/h remifentanil hydrochloride (Daiichi-Sankyo Propharma Co., Ltd.) and 25–50 mg/kg/min lidocaine (Aspen Japan, Tokyo, Japan) was also performed for intraoperative analgesia. A 0.3 mg/kg of morphine hydrochloride (Takeda Pharmaceutical Co. Ltd., Osaka, Japan) was administered intramuscularly before and after surgery. After surgery, continuous intravenous administration of 1.25–5.0 mg/kg/h remifentanil hydrochloride (Daiichi-Sankyo Propharma Co., Ltd.) was maintained for postoperative analgesia.

### Surgical procedure

**Figure 1:**
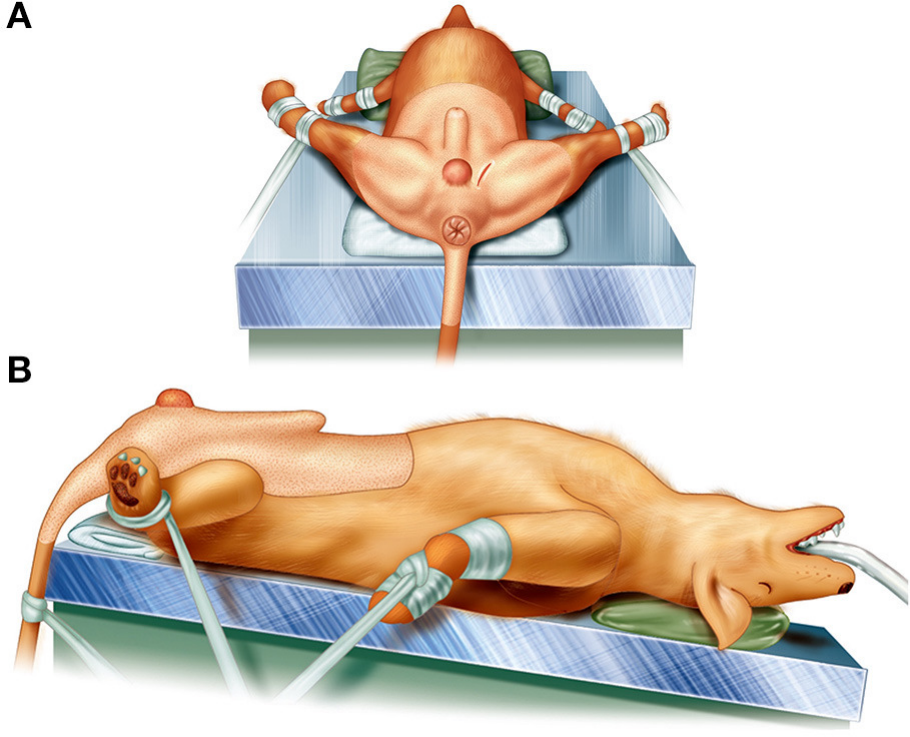
Illustrations of patient's position for perineal herniorrhaphy using the pedunculated tunica vaginalis communis. The patient was held in the Trendelenburg position at the edge of the operating table, the hind limbs were pulled sideways, and the tail was fixed so as to hang down. **(A)** The caudal view of the position and **(B)** the left side view.

The skin of the abdomen, perineum, and scrotum of each dog were prepared for aseptic surgery. All canine patients were held in the Trendelenburg position at the edge of the operating table, with the hind limbs pulled sideways and the tail hanging down (Figure 1).

**Figure 2:**
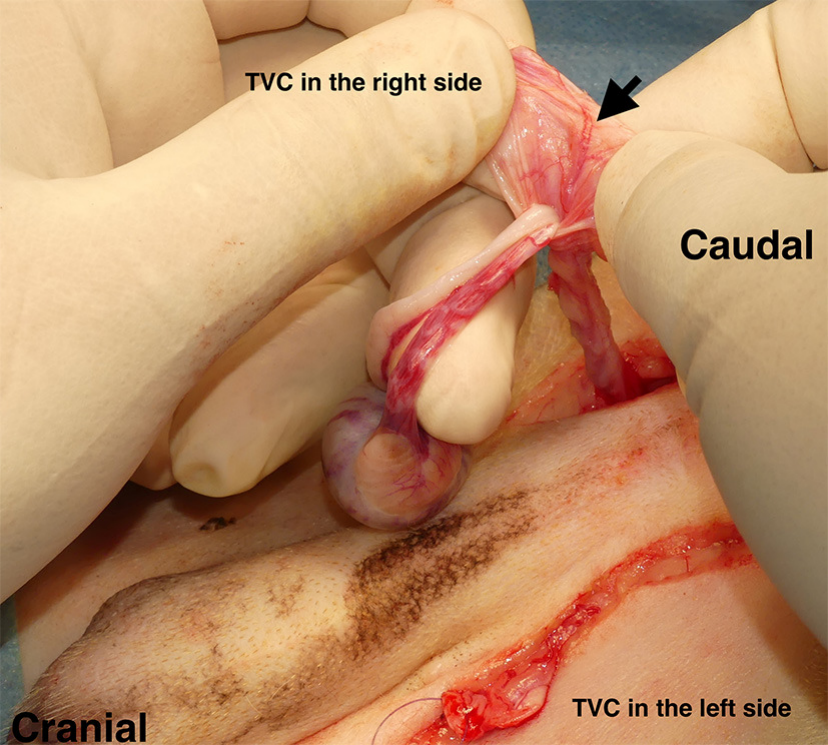
Castration prior to perineal herniorrhaphy. Open castration was first performed by making an incision in the skin close to the inguinal region. After the open castration, the remaining tunica vaginalis communis (TVC) on each side was isolated with preserved blood flow. Black arrow: arteriole branches of the cremasteric aorta.

A skin incision was made close to the right side of the inguinal region. Subsequently, open castration surgery was performed with sufficient exposure of the TVC. Additionally, castration of the left side was performed in the same manner: arteriole branches of the cremasteric aorta were preserved for the inherent blood supply to the TVC (Figure 2). After the isolation of TVC, the stay suture was placed at the tip of each TVC. Colopexy was performed when the caudal displacement of the anus due to fecal retention was observed based on clinical findings and lateral abdominal radiographs. Cystopexy was performed when the caudal displacement of the bladder and prostate was observed on lateral abdominal radiography. When colopexy and/or cystopexy were added, an abdominal skin incision for caudal abdominal celiotomy was made avoiding the penis, and the colon and bladder were sutured and fixed to the left and right abdominal wall, respectively, with a 2-0 or 3-0 polydioxanone suture (PDS II^®^; Johnson & Johnson, New Brunswick, NJ, USA), as needed.

**Figure 3:**
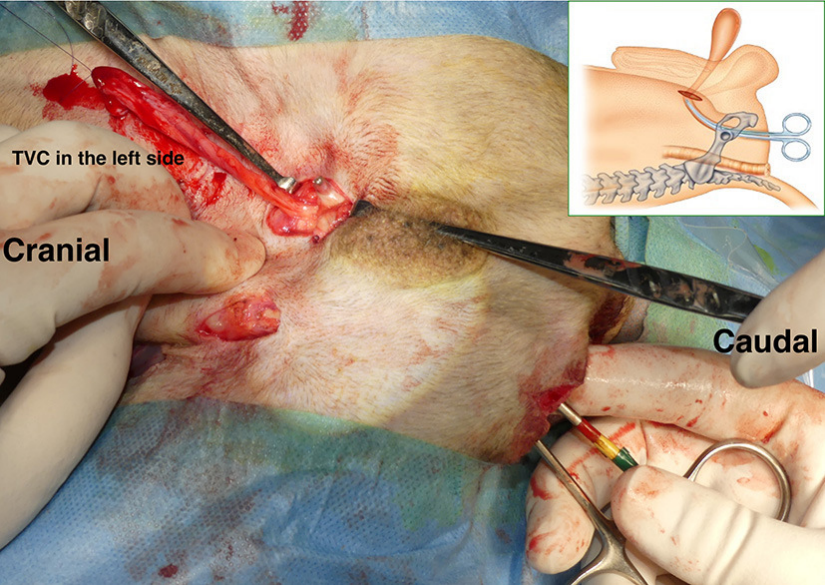
Transposition of pedunculated tunica vaginalis communis (TVC) into the hernial foramen. After making the tunnel with an index finger, we put the forceps from the hernial foramen into the tunnel and advanced it toward the inguinal ring. The tips of the forceps penetrated outward from the abdominal wall near the inguinal ring. The stay suture placed at the tip of the TVC was then grasped with the forceps, and the TVC was transposed from the inguinal ring through the pelvic cavity into the hernia. An illustration shows the forceps passing through the pelvic cavity from the hernial foramen.

With the dog in the Trendelenburg position, a skin incision was made on the affected part of the PH, on one or both sides, starting over the ischial tuberosity and extending dorsally. The subcutaneous tissues were dissected to confirm PH. Atrophy of the pelvic septal muscles was checked, and the anatomical positions of the sacrotuberous ligament, internal obturator muscle, ischium, and external anal sphincter muscle were confirmed visually or by palpation. The index finger was inserted into the abdominal cavity through the PH. The position of the inguinal ring was confirmed on the inside of the abdominal cavity using the index finger. As the index finger created a tunnel from the PH through the abdominal cavity to the inguinal ring, a right-angled forceps was inserted from the PH, which passed through the tunnel created by the index finger, and angled ventrally toward the inguinal ring. The tips of the forceps penetrated outward from the abdominal wall near the inguinal ring (Figure 3). The stay suture placed in the TVC was grasped by the forceps and pulled through the abdominal wall to the PH. Therefore, the pedunculated TVC was transposed from the inguinal ring through the pelvic cavity into the PH.

**Figure 4:**
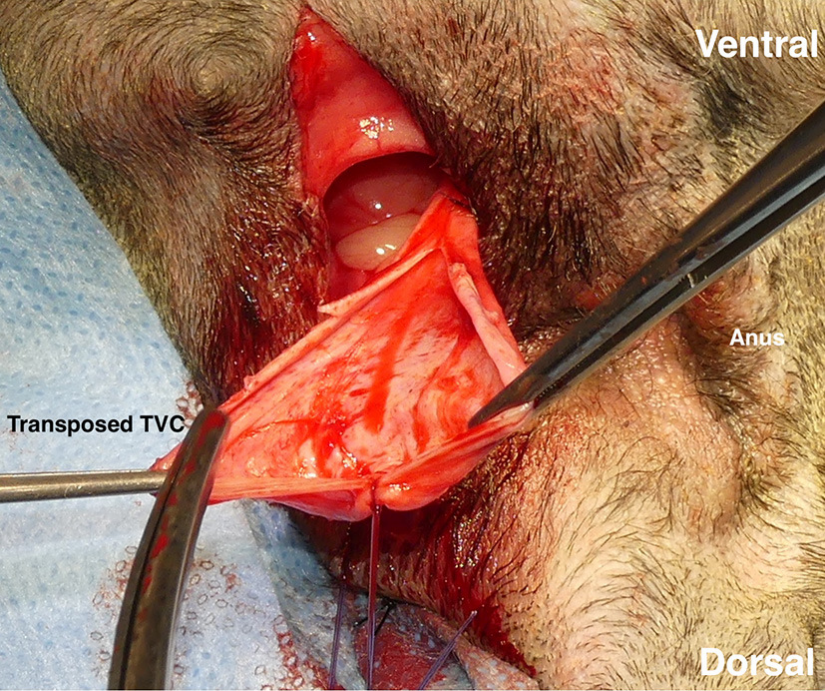
Tunica vaginalis communis (TVC), which was transposed to the hernial foramen. After confirming the preservation of its blood vessels, a vertical incision was made to expand the TVC into a fan shape.

**Figure 5:**
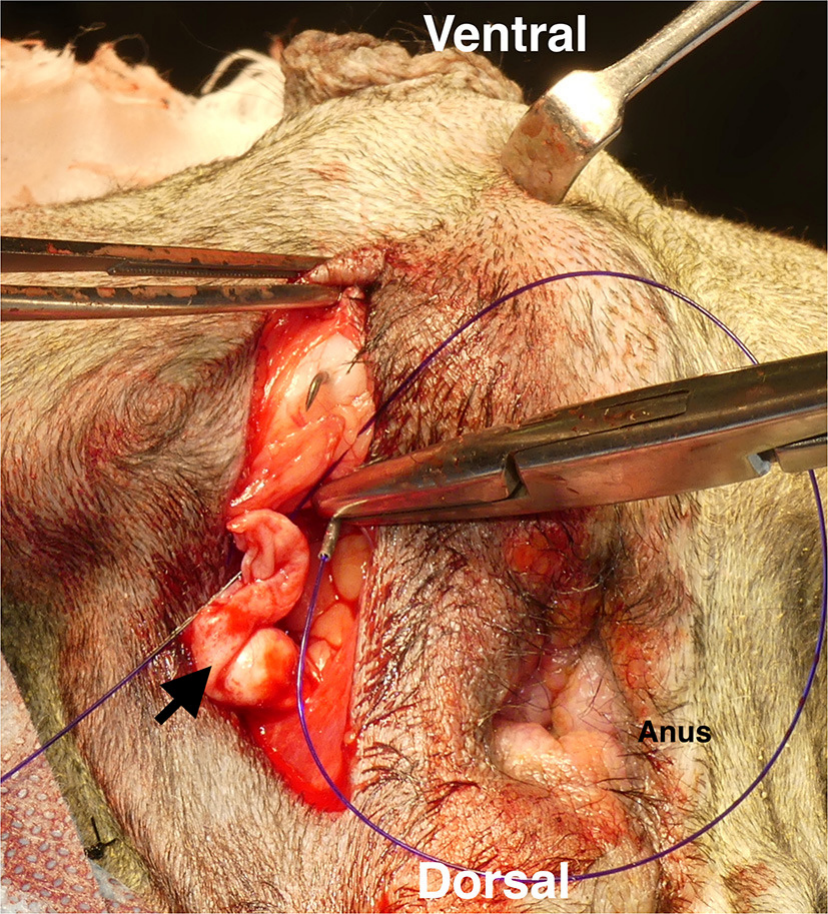
Ligating the transposed tunica vaginalis communis (TVC) with the internal obturator muscle. First, TVC was ligated with sacrotuberous ligament at three-four sites. It was then ligated with the internal obturator muscle at three-four sites. Black arrow: transposed TVC.

**Figure 6:**
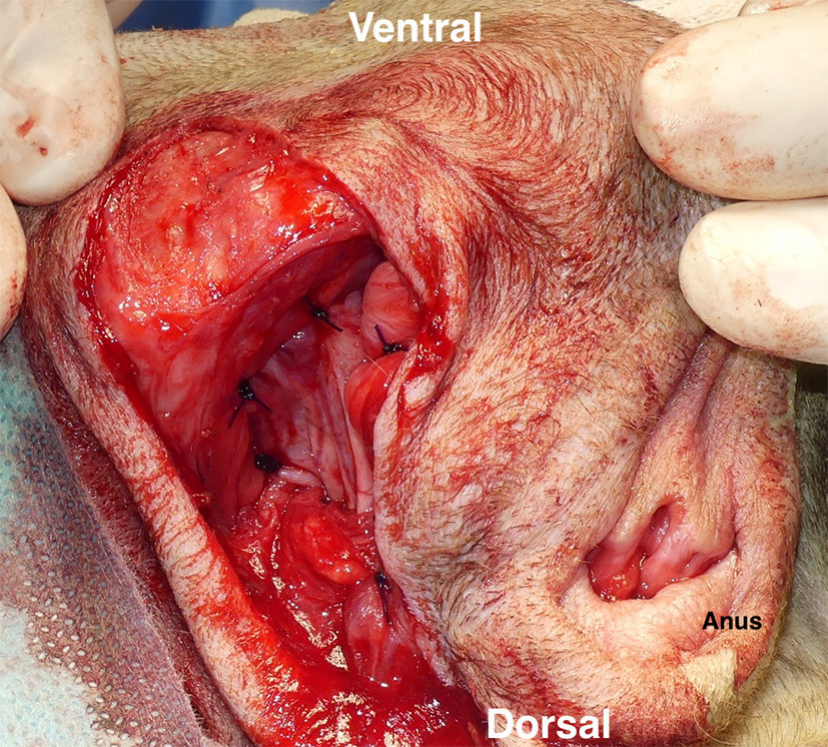
Coverage of a right-sided perineal hernia with the expanded tunica vaginalis communis (TVC). The TVC was sutured with the external anal sphincter muscle on the medial side, the sacrotuberous ligament on the lateral side, and the internal obturator muscle and the ischial periosteum on the ventral side.

A vertical incision of the pedunculated TVC was made to open it to a fan shape (Figure 4). When the TVC graft was larger relative to the hernial foramen, it was folded to increase the strength. When the PH was bilateral, each ipsilateral TVC was used. When the PH was unilateral, both TVCs were used for the affected side. When the TVC on the opposite side of the PH was used, the index finger was inserted from the PH and carefully advanced to the contralateral inguinal ring through the pelvic cavity to create the tunnel. The contralateral TVC was transposed to the PH by the right-angled forceps passing through the tunnel the same way the ipsilateral TVC was transposed. The TVCs were sutured to the sacrotuberous ligament with three-four 2-0 polydioxanone suture materials (PDS II^®^; Johnson & Johnson). Subsequently, they were sutured to the internal obturator muscle (Figure 5) and the ischial periosteum with three-four of the same materials. Finally, the TVCs were sutured to the external anal sphincter and the remaining coccygeal muscle with three-four of the same materials (Figure 6), and the repair of PH was completed. The subcutaneous tissues were closed with 3-0 poliglecaprone 25 (Monocryl^®^; Johnson & Johnson), and the perineal skin was routinely closed. In case of colopexy and/or cystopexy, the abdominal incision was routinely closed with 2-0 polydioxanone (PDS II^®^; Johnson & Johnson). The subcutaneous tissues and skin incisions of the abdominal and inguinal areas were then routinely closed.

### Postoperative progress

During hospitalization, the general condition of each dog was checked; the presence or absence of serous fluid, dehiscence, and local infection in the surgical wound were evaluated as postoperative complications. In addition, physical examination, CBC, serum chemistry, and abdominal radiography were performed during hospitalization, as needed.

After discharge, physical examination, rectal digital examination, CBC, serum chemistry, and abdominal radiography were performed on every consultation day. The presence or absence of recurrence was checked. The postoperative prognosis of all patients was checked based on information from medical records or telephone records from referring veterinarians or the owners.

## Results

### Signalment

The results are summarized in Supplementary Table 1. Of the eight dogs, the most common breeds were Toy poodle (three cases), Mix-breeds (two cases), followed by Miniature dachshunds, Shih Tzu, and Yorkshire terrier (one each). The median age of the dogs was 10.5 years (range, 9–13 years), and the median weight was 4.9 kg (range, 1.6–12.3 kg).

### Clinical signs

Six dogs had unilateral PH (one on the left and five on the right side), and two dogs had bilateral PH. PH-related clinical symptoms, including tenesmus, were observed in five dogs (63%), of which two dogs (25%) needed the owner's assistance due to difficulty in defecation. Until surgery, supportive treatment was performed by administering a fecal softener, such as lactulose, in three cases. No clinical signs related to urination were observed in any dog.

### Intraoperative findings

The median period from symptom onset to surgery was 2.5 months (range, 1–25 months). Perineal herniorrhaphy using the pedunculated TVC was feasible in all dogs; colopexy alone was performed in one dog, cystopexy alone was performed in two dogs, both procedures were performed in three dogs, and neither was performed in two dogs. The median operation time was 105.5 min (range, 46–149 min) in unilateral PH, whereas the operation time was 92 and 122 min in two dogs with bilateral PH.

### Postoperative progress

The median hospitalization period was 9.5 days (range, 3–24 days), and the median follow-up period in our hospital was 395.5 days (range, 15–2,294 days). Two dogs (25%) had suspected local infection during the follow-up period on postoperative day (POD) 6. In one dog, purulent drainage was observed from the perineal incision site; therefore, bacterial infection was suspected. Fosfomycin calcium hydrate (Meiji Seika Pharma Co., Tokyo, Japan) was chosen based on the results of bacterial culture and was intravenously administered at a dose of 30 mg/kg twice a day for 6 days, followed by oral administration for 18 days. Purulent drainage was similarly observed in another dog. However, bacterial culture was not performed, and fosfomycin calcium hydrate (Meiji Seika Pharma Co.) was administered orally at 30 mg/kg twice a day for 24 days. The antibiotic administrations were determined based on each dog's physical and hematological examinations. In both cases, the local infection was curable after the medication. No major short-term complications, including dehiscence and sepsis, were observed during the hospitalization period in all dogs.

After discharge, recurrence was observed in one dog (13%). In this case, a rectal digital examination revealed the recurrence of PH and rectal enlargement on POD 136. The owner of the dog did not opt for revisional surgery. No other major postoperative complications were observed in the residual seven dogs. Preoperatively observed tenesmus could be postoperatively controlled by a high fiber diet and medical management with the laxative lactulose (Teva Takeda Pharma Ltd.) and/or mosapride citrate hydrate (Pronamide^®^; DS Pharma Animal Health Co., Ltd., Osaka, Japan). No clinical symptoms related to urination were observed in all dogs. According to the owners' or referring veterinarians' responses on the questionnaire, none of the dogs seemed to be negatively affected by the surgery, and the functional outcome and quality of life were good in all dogs.

## Discussion

Our study demonstrated that the pedunculated TVC graft could be used for perineal herniorrhaphy in intact male dogs. There are many techniques for the treatment of PHs, and as many of the traditional techniques have been developed further, surgical outcomes have improved. The most common technique, internal obturator muscle transposition herniorrhaphy, was reported to have a no postoperative complications rate of 51.2%−93% and a recurrence rate of 0–27.4% (7, 8). In addition, the semitendinosus muscle transposition herniorrhaphy, which can be performed in severe cases, was reported to have a postoperative complication rate of 21.4% and a recurrence rate of 14.3% (9). In our study, no major complications were observed, and postoperative local infections and tenesmus were curable with medication; other studies have reported the same complications. Therefore, this procedure with the pedunculated TVC graft is suggested to be an alternative option for the repair of PH in intact male dogs.

Perineal hernia is particularly common in intact males, and in most cases, castration is performed at the same time as reconstruction of the pelvic diaphragm components. Hence, a surgical procedure using autologous TVC could be possible for many PH cases. In reconstruction using autologous tissues, semitendinosus muscle translocation is reportedly associated with a postoperative infection rate of 40% due to the large incision required (21); thus, muscle flaps carry a risk of postoperative infection. In addition, the autogenous fascia lata graft and semitendinosus muscle translocation resulted in postoperative lameness (9, 10). Lameness improves in most cases; however, these procedures may result in donor site dysfunction in the postoperative period. In our study, the procedure was expected to lead to reduced postoperative invasion compared to the commonly performed muscle transposition.

In a previous report wherein a TVC autologous free graft was implanted, no postoperative recurrence or complications were observed in all seven dogs (19). In another report, after a surgical procedure involving internal obturator muscle transposition followed by implantation of an additional patch with an autologous free TVC graft, one case showed recurrence, and two cases showed postoperative complications, out of a total of nine cases (20). In our case series, the postoperative recurrence rate was 13%, which is comparable to that in the other two reports. We hypothesized that the pedunculated TVC graft is more effective for perineal herniorrhaphy than the free graft due to the preservation of blood supply. In a report of abdominal wall reconstruction in rats using the ECM, angiogenesis in the early stage after transplantation promoted cell infiltration and led to a decrease in the postoperative recurrence rate (22). However, postoperative histological findings were not investigated in this study. It may be necessary to compare the postoperative histological findings after TVC transposition with the postoperative course in the future.

The pedunculated TVC includes a structure that continues from the inguinal ring to the perineum; the TVC is inverted and guided from the inguinal ring to the perineum and is then sutured to the pelvic diaphragm components. The connection of the pedunculated TVC between the inguinal ring and the pelvic diaphragm components is believed to potentially strengthen the reconstructed perineal area against the abdominal and pelvic pressure: i.e., the connection might prevent the caudal displacement of the reconstructed perineal area, which can result in the recurrence of PH. However, as postoperative anatomical and histological investigations were not performed in our study, further studies are necessary to evaluate the postoperative anatomical and histological findings after TVC transposition and the postoperative course. However, as postoperative anatomical findings were not investigated in this study, further studies are necessary to compare the postoperative anatomical findings after TVC transposition with the postoperative course.

One of the most common complications is the recurrence of PH. In our study, recurrence occurred in one case (13%); recurrence was suspected on POD 136 based on the results of a rectal examination. The cause of recurrence was thought to be the shrinkage of the transposed TVC tissue over time. When observing the shrinkage of the mesh surface compared with the original size, the ECM shows significantly more shrinkage compared to the polypropylene mesh (23). Compared with the autologous TVC free graft, the pedunculated TVC graft used in our study may retain its strength and prevent shrinkage due to the preservation of blood supply. However, our study did not investigate the postoperative change in the transposed TVC tissue. Further studies are required to clarify whether the strength of pedunculated TVC tissue is related to recurrence.

In our study, colopexy and/or cystopexy were performed in some cases prior to PH reconstruction. A previous study described that colopexy and cystopexy are not associated with an increased rate of postoperative complications and have little effect on long-term outcomes in dogs with PH (24). Our study also could not demonstrate the impact of these techniques on the surgical outcome. However, the caudal displacement of the anus, bladder, and prostate might impede the reconstruction of PH using the pedunculated TVC. In such cases, we observed that colopexy and cystopexy could facilitate the fixing of sutures of the transposed TVC to the pelvic septal structures (sacrotuberous ligament, internal obturator, and external anal sphincter muscle). Therefore, we considered that colopexy and cystopexy might be useful in canine PH with caudal displacement of the anus, bladder, and prostate. However, further investigations are warranted to clarify the improvement of surgical outcomes by concurrent colopexy and/or cystopexy.

Perineal herniorrhaphy was feasible in the Trendelenburg position in our study, although it is generally performed in the sternal position. Castration surgery is indispensable for PH treatment in intact male dogs. In addition, colopexy and/or cystopexy are performed in some cases. Hence, surgical repair in the Trendelenburg position eliminates the need for repositioning. Compared with the sternal position, the intra-abdominal organs are moved cranially in the Trendelenburg position, preventing the organs from being displaced in the perineal region during surgery, thus securing the field of view of the perineal surgical wound, and shortening the operation time. Therefore, the Trendelenburg position of canine patients with PH may be useful for perineal herniorrhaphy.

A major limitation of our study was the small sample size. Our surgical technique is not applicable for females, castrated males, and patients with testicular tumorigenesis. Another limitation is the inconsistency of the postoperative examination period. In the future, we will need to collect more cases and conduct an additional long-term (2 years or more) follow-up of surgical procedures in which TVC is applied to PH in dogs to examine possible postoperative complications.

## Conclusions

Our study suggests that the herniorrhaphy technique using the pedunculated TVC could be an alternative option for the repair of PH in intact male dogs.

## Data availability statement

The original contributions presented in the study are included in the article/Supplementary material, further inquiries can be directed to the corresponding author.

## Ethics statement

The animal study was reviewed and approved by Ethical Committee of Nihon University Animal Medical Center. Written informed consent was obtained from the owners for the participation of their animals in this study.

## Author contributions

ST contributed to developing and establishing the surgical procedure. KA contributed to developing and establishing the surgical procedure, designing, and planning the study and wrote and reviewed the manuscript. TH contributed to designing and planning the study and wrote the manuscript. KI, OY, NS, KaT, MS, and KeT contributed to supporting the study. All authors participated in the surgeries and peri-operative management. All authors contributed to the article and approved the submitted version.

## Conflict of interest

The authors declare that the research was conducted in the absence of any commercial or financial relationships that could be construed as a potential conflict of interest.

## Publisher's note

All claims expressed in this article are solely those of the authors and do not necessarily represent those of their affiliated organizations, or those of the publisher, the editors and the reviewers. Any product that may be evaluated in this article, or claim that may be made by its manufacturer, is not guaranteed or endorsed by the publisher.

## Abbreviations

PH: perineal hernia
TVC: tunica vaginalis communis
ECM: extracellular matrix
CBC: complete blood count
POD: postoperative day.
